# Short-Term Ketogenic Diet Improves Abdominal Obesity in Overweight/Obese Chinese Young Females

**DOI:** 10.3389/fphys.2020.00856

**Published:** 2020-07-28

**Authors:** Zhaowei Kong, Shengyan Sun, Qingde Shi, Haifeng Zhang, Tomas K. Tong, Jinlei Nie

**Affiliations:** ^1^Faculty of Education, University of Macau, Macao, China; ^2^Institute of Physical Education, Huzhou University, Huzhou, China; ^3^School of Health Sciences and Sports, Macao Polytechnic Institute, Macao, China; ^4^College of Physical Education, Hebei Normal University, Shijiazhuang, China; ^5^Department of Physical Education, Hong Kong Baptist University, Hong Kong, China

**Keywords:** low-carbohydrate, subcutaneous fat, weight loss, cardiorespiratory fitness, leptin

## Abstract

The purpose of this study was to examine the effects of a short-term ketogenic diet (KD) on body composition and cardiorespiratory fitness (CRF) in overweight/obese Chinese females. Twenty young females [age: 21.0 ± 3.7 years, weight: 65.5 ± 7.7 kg, body mass index (BMI): 24.9 ± 2.7 kg⋅m^–2^] consumed 4 weeks of a normal diet (ND) as a baseline and then switched to a low-carbohydrate, high-fat, and adequate protein KD for another 4 weeks. With the same daily caloric intake, the proportions of energy intake derived from carbohydrates, proteins, and fats were changed from 44.0 ± 7.6%, 15.4 ± 3.3%, 39.6 ± 5.8% in ND to 9.2 ± 4.8%, 21.9 ± 3.4%, and 69.0 ± 5.4% in KD. The results showed that, without impairing the CRF level, the 4-week KD intervention significantly reduced body weight (−2.9 kg), BMI (−1.1 kg⋅m^–2^), waist circumference (−4.0 cm), hip circumference (−2.5 cm), and body fat percentage (−2.0%). Moreover, fasting leptin level was lowered significantly, and serum levels of inflammatory markers (i.e., TNF-α and MCP-1) were unchanged following KD. These findings suggest that KD can be used as a rapid and effective approach to lose weight and reduce abdominal adiposity in overweight/obese Chinese females without exacerbating their CRF.

## Introduction

The incidence of obesity and related diseases is increasing rapidly and is a major health challenge faced by both developed and developing countries (Roberts and Barnard, 2005). In contrast to the accumulation of peripheral subcutaneous fat, excessive accumulation of adiposity in abdominal viscera has a more direct association with obesity-related complications, including diabetes, metabolic syndrome, hepatic steatosis, aortic plaque (Neeland et al., 2013), and abnormal activity of the autonomic nervous system (Triggiani et al., 2019). The beneficial effects on metabolic syndrome parameters resulting from visceral fat reduction were greater than those induced by subcutaneous fat reduction (Park and Lee, 2005).

Although the conventional dietary guidelines for weight loss recommend low fat intake and calorie restriction resulting in a negative energy balance (Seagle et al., 2009), accumulating studies mainly from Western countries have shown that the low-carbohydrate diet approaches are effective in fighting obesity and improving cardiometabolic health (Sharman et al., 2004; Volek et al., 2004a; Brinkworth et al., 2009a; Gu et al., 2013; Sun et al., 2019). Generally, low-carbohydrate diets are considered to contain <100 g/day or <30% of energy from carbohydrates (Sumithran and Proietto, 2008), and very low carbohydrate diets (or called ketogenic diet, KD) are characterized by a daily intake of less than 50 g of carbohydrates (Paoli et al., 2013). It has been revealed that, in comparison with low-fat diets, low-carbohydrate diets resulted in greater weight reduction (Sharman et al., 2004; Volek et al., 2004a) and led to more favorable alterations in blood lipids (Sharman et al., 2004; Brinkworth et al., 2009a) and glucose regulation (Sharman et al., 2004). Undeniably, the evidence regarding the effectiveness of low-carbohydrate diets in weight reduction and cardiometabolic health improvement is strong; however, it is less popular in China. It is noteworthy that people in China and Western countries have different dietary patterns and food preferences (Popkin and Gordon-Larsen, 2004; Zhai et al., 2009); thus, their attitude and acceptance of low-carbohydrate diets may be distinct. Moreover, in limited studies involving the Chinese population, researchers have put an overemphasis on calorie restriction (Gu et al., 2013; Liu et al., 2013; Sun et al., 2019), making it difficult to specify whether the low-carbohydrate diet-induced weight-loss effects are a result of reduction in energy intake or changes in macronutrient proportions. Furthermore, several previous studies pointed out that very low-carbohydrate diets (i.e., KD) seemed particularly effective in reducing subcutaneous and visceral fat mass (Gu et al., 2013; Valenzano et al., 2019) as well as trunk fat mass (Volek et al., 2004a). Therefore, it is necessary to assess whether non-calorie-restriction KD dietary patterns are also useful and feasible for the large overweight/obese population in China.

During KD intervention with strictly restricted carbohydrate content (usually <50 g/day), the typical metabolic changes in individuals without preadaptation are lowering muscle glycogen restoration rates and decreasing glycolytic-enzyme activities (Chang et al., 2017), and higher serum concentrations of non-esterified fatty acids and ammonia following KD may also contribute to central fatigue (Fiorenzo et al., 2020). As a result, several previous studies revealed that the KD intervention might lead to early development of central fatigue (Chang et al., 2017) and further impaired cardiorespiratory fitness (CRF) (Okeeffe et al., 1989; Urbain et al., 2017) or exercise performance (Pilis et al., 2018). However, the findings are not always consistent. After adaption, the aerobic and anaerobic exercise capacity of athletes seems to be unaffected by KD (Zajac et al., 2014; Fiorenzo et al., 2020). CRF is a strong independent indicator for cardiovascular diseases and all-cause mortality (Kodama et al., 2009; Kaminsky et al., 2013). The necessity of evaluating and intervening CRF are highlighted by the American Heart Association to reduce risk factors of cardiovascular disease and promote overall cardiovascular and general health (Kaminsky et al., 2013). Any diet that potentially impair someone’s CRF level and the ability to adhere to an exercise regime would be of great concern. Therefore, a maximal incremental exercise test was adopted as one of the main outcome measurements in the present study. The objective was first to examine the efficacy of a 4-week non-calorie-restriction KD on body composition and the impact of KD on CRF in the overweight/obese Chinese females. In addition, serum concentrations of appetite control hormones and inflammatory biomarkers were assessed in this study.

## Materials and Methods

### Subjects

This study was approved by the Panel on Social Science & Humanities Research Ethics of the University of Macau (RC Ref. no. MYRG2017-00199-FED). Recruitment notices, including research purposes, qualifying criteria, and brief research procedures, were released to campus bulletin boards and emails to recruit overweight/obese but healthy women interested in this study. The inclusion criteria were (1) overweight or obese defined as body mass index (BMI) ≥ 23 kg⋅m^–2^ (World Health Organization, 2000), (2) between 18 and 30 years old, (3) body weight remained stable in the past 6 months (variation within 5% of body weight), (4) healthy (without any endocrine, metabolic, osteoarticular, gastrointestinal, hepatic, renal, or cardiovascular diseases), (5) sedentary lifestyle (not participant in regular physical activity). Subjects who meet any of the following items were excluded: comfortable drinking alcohol, smokers, participate in structured training programs or specific diet programs at the time of recruitment, have physical barriers to exercise, take any prescribed medicines or nutritional supplements, or have respiratory problems or eating disorders. After the screening process, 24 eligible overweight/obese but healthy young females (19–25 years old) were included. All subjects provided written informed consent before being formally involved. Four subjects withdrew for different reasons; 20 subjects who completed the normal diet (ND) period, KD period, and all three outcome measurements were included in final data analysis.

### Experimental Design

**FIGURE 1:**
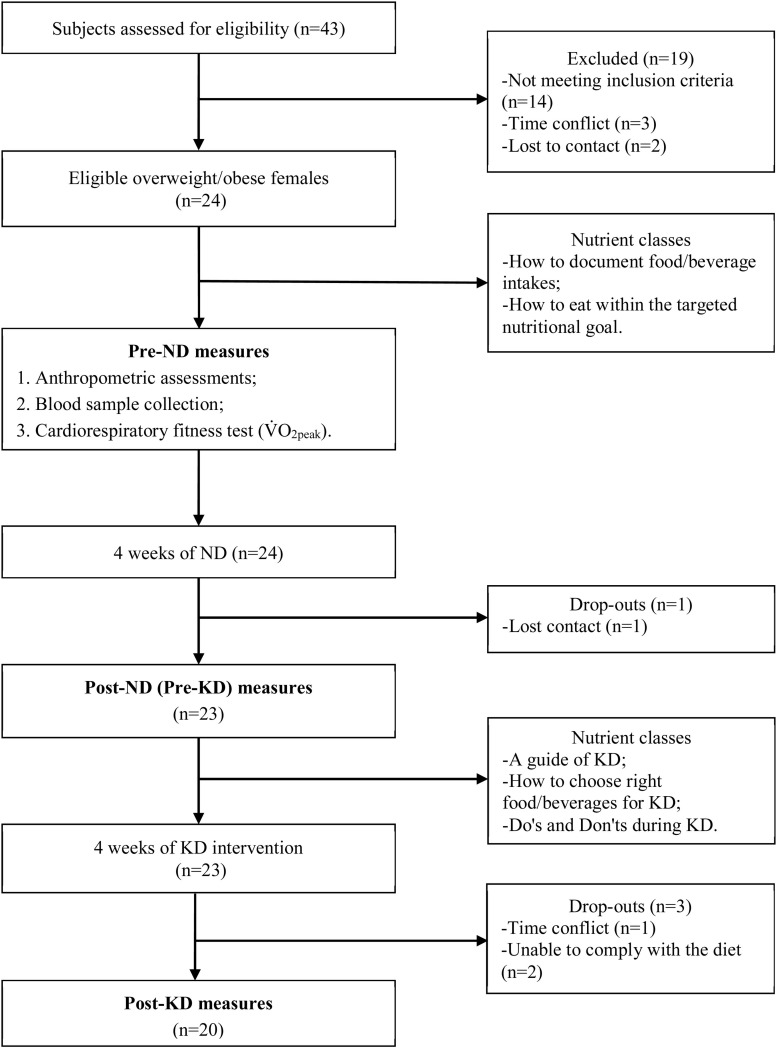
Flow-chart of the study.

This study was performed in the following order: a preparation phase with two nutrition classes, the first measurement of main outcome variables (including anthropometric assessments, blood sampling, and a maximal incremental exercise test) prior to ND, a 4-week ND (28 days), the second outcome measurements after ND, another three nutrition classes about KD, a 4-week KD intervention (28 days), and the last outcome measurements after KD intervention (Figure 1).

During the preparation period, subjects received two nutrition classes on how to document food/beverage intake and how to eat within the targeted nutritional goal. Digital scales, food measuring instruments, and detailed instructions and individual consultation were given to all subjects so that they could accurately record the weight and amounts of food/beverage intake. Because the three measures of the main outcome variables were performed within the same phase of each subject’s menstrual cycle (i.e., the luteal phase), we asked them to recall and provide their menstrual phases over the past 3 months in the preparation period. Then, the days of outcome measurements were calculated individually according to their self-reported menstrual phases, and the menstrual phases for the next 3 months were estimated.

The first measurement of main outcome variables was conducted 2–5 days prior to ND, then a 4-week ND was performed as a control period, and 3-day food diaries (2 weekdays and 1 weekend day) were kept by the subjects during this period. The second outcome measurements were completed within 72–96 h following the last day of ND. Meanwhile, another three nutrition classes on KD were offered, namely a guide to KD, how to choose the right food and beverage for KD, and dos and don’ts during KD. A handout that outlines the main aspects of KD and a specific list of suitable foods, beverages, cooking recipes, and sample meals for KD was also provided. Thereafter, subjects consumed 4 weeks of low-carbohydrate, high-fat KD according to the instruction and kept 3-day diet diaries in the same way as ND. The last outcome measurement was completed between 72 and 96 h following the last day of KD.

### Diet Intervention

During the 4-week ND period, subjects maintained their habitual diet and then switched to KD for another 4 weeks, in which they had ∼10% of daily energy intake from carbohydrates (approximately 50 g/d), ∼65% of energy intake from fats, and ∼25% of the rest from protein.

Subjects could choose low-carbohydrate foods/beverages according to their own preferences and were required to have low-carbohydrate foods/beverages and restrict or avoid foods/beverages with high carbohydrate content. Foods/beverages appropriate for KD included all kinds of fat, oils, all kinds of meat (e.g., pork, beef, fowl such as chickens and ducks), eggs, seafood, cheese, non-starchy and green vegetables, nuts/seeds, water, and low-carbohydrate beverages (e.g., green/red tea, black coffee). Although the types of fat from saturated or unsaturated sources were not restricted, we encouraged subjects to add five tablespoons of olive oil to their daily diet. Foods/beverages to avoid during KD included rice, cereals, products made of flour (e.g., bread, noodles, cakes), sugar, desserts, sweets, honey, beans, corns, starchy vegetables, fruits (with the exception of blueberry, lemon, and avocado), milk, yogurt, soft drinks, juices, and alcoholic beverages.

To assure subjects’ adherence to KD, we required them to measure urinary ketones every day and record 3-day food diaries (2 weekdays and 1 weekend day) during the experimental period. Reagent strips (UROPAPER, Suzhou First Pharmaceutical Co., Ltd., Suzhou, China) were provided to all subjects for self-assessment of daily urinary ketones, which was performed in the early morning or after dinner (Urbain and Bertz, 2016). The minimum detectable amount of the reagent strip was 10 mg/dL, and the rate of concordance was 95.1% with clinical diagnosis (Ishizaki et al., 2016). Moreover, 3-day food diaries were kept by all subjects for 8 weeks (the 4-week ND period and the 4-week KD period). Thorough instructions on how to estimate portion sizes and record food/beverages intake on food composition tables were given to all subjects in advance. Subjects were asked to report to the laboratory every week to assess changes in body weight and hand in the logbook with food diaries. Energy intake and macronutrient composition were calculated by the same dietician using the nutrition analysis and management system (NRISM, version 3.1, China). Diet compliance was evaluated based on the results of the urinary ketones and food diaries, and subjects received follow-up dietary advice and counseling individually from the dietician. Constant assistance was provided throughout the study; if subjects had any queries, problems, or feedback relating to the experiment, they could contact the researchers via phone, WeChat, e-mail, or meet in person and get answers immediately.

In addition to the targeted intervention, subjects were required to maintain their habitual daily routines and not take extra exercise throughout the study period. Meanwhile, validated pedometers (Yamax SW-200 digiwalker, Japan) were provided to all subjects to assess their daily physical activities. Each subject received a logbook with a calendar to record daily food intake, daily urinary ketone test results, daily physical activities (in steps), and any adverse side effects or symptoms of the intervention.

### Measures of Main Outcome Variables

#### Blood Profiles

Blood samples were collected at the same phase of each subject’s menstrual cycle (i.e., the luteal phase) at different measurement time points. Strenuous physical activity, caffeine, and alcohol were prohibited for 48 h before blood sample collection. Subjects arrived at the laboratory at around 7 a.m. under the condition of fasting overnight (>10 h), and 5 ml blood was drawn from the cubital vein by a certificated nurse using a serum separation tube. The blood samples were left for clotting at room temperature for 1 h and then centrifuged at 3000 rpm for 5 min; serum was separated and immediately frozen at −80°C for later analysis.

Leptin, ghrelin, tumor necrosis factor alpha (TNF-α), and monocyte chemoattractant protein-1 (MCP-1) were measured using the EMD Millipore Milliplex MAP immunoassay (Merck KGaA, Darmstadt, Germany). All blood samples were measured in standard procedures in accordance with the manufacturer’s instructions (KingMed Diagnostics Co., Ltd., Guangzhou, China), and were conducted at the end of the study to minimize variability.

#### Anthropometric Assessments

Anthropometric assessments were conducted the same morning after blood samples were taken (without breakfast). Height and weight were measured using a wall-mounted stadiometer and an electronic scale in a standard manner (barefoot and wearing light clothes), and the values were recorded to the nearest 0.1 cm and 0.1 kg, respectively. Body weight (kg) divided by square height (m^2^) was calculated as BMI (in kg⋅m^–2^). Waist circumference (WC) was measured at the intermediate position between the upper edge of the iliac crest and the lower edge of the 12th rib while the subject was breathing out gently; hip circumference (HC) was determined as the maximum circumference over the buttocks; the WC and HC values were recorded to the nearest 0.5 cm. Waist-to-hip ratio (WHR) was calculated as WC divided by HC (both in cm). Skinfold thickness measurements were taken on the right side of the body using a Harpenden skinfold caliper (British Indicators Ltd., St Albans, Herts) with the subjects in a standing posture. Three sites were selected for skinfold thickness measurements, including (1) triceps: in the middle between the olecranon and the tip of the acromion, over the midpoint of the muscle belly with the upper arm suspended vertically; (2) anterior superior iliac spine: the highest point of the pelvis that can be touched from the front, the skinfold was lifted along the iliac crest; and (3) thigh: at the midpoint of the groin and knee, on the front side of the thigh. At these three sites, the skinfold was firmly pinched between the thumb and forefinger and gently pulled away from the underlying tissues before the caliper was applied for measurement. The opening width was read off on a scale incorporated in the apparatus and recorded to the nearest 0.1 mm. BF% was calculated using the following equation: BF% = 100 × (4.95/body density−4.5) (Siri, 1956), and body density = 1.0994921−0.0009929 × (sum) + 0.0000023 × (sum)^2^ −0.0001392 × age (Jackson et al., 1980), where sum refers to the sum of skinfold thickness measured at the above three sites. All anthropometric measurements were performed by the same investigator using the same instrument.

#### Maximal Incremental Exercise Test

The maximal incremental exercise test was taken to determine the CRF level. After a brief warm-up, subjects started to pedal on an electric-braked cycle ergometer (Monark 839E, Vansbro, Sweden) with an initial workload of 50 W. The workload was increased by 25 W in 3 min intervals until the subjects reached their volitional exhaustion and then recovered at 25 W for 3 min. The pedaling speed was maintained at 60 ± 5 rpm throughout the test. During the V̇O_2__peak_ test, respiratory gases were continuously assessed using a gas analyzer (Vmax Encore System, CareFusion Corp., San Diego, CA, United States). V̇O_2__peak_ was calculated as the largest oxygen consumption value averaged over 15 s of the last exercise stage (Rossiter et al., 2006).

### Statistical Analysis

Statistical analyses were conducted using the PASW software (Release 22.0; IBM, NY, United States). Prior to the main statistical analyses, the Shapiro–Wilk test was performed to confirm whether the outcome variables were normally distributed. One-way repeated-measures analysis of variance (ANOVA) was performed to detect the differences in body composition; CRF level; blood profiles among the three time points (pre-ND, post-ND, and post-KD); and the differences in dietary energy intake, macronutrient composition, and daily physical activities among the eight time points measured across the study (4 weeks of ND and 4 weeks of KD). A paired-sample *t*-test was used to compare the differences in changes of main outcome variables after ND and KD. Pearson’s correlation tests were performed to examine the associations between body composition variables and hormones (i.e., leptin and ghrelin). Partial η^2^ values were used to assess the effect sizes of the main and interaction effects; η^2^ was considered small if <0.06 and large if >0.14 (Kirk, 1996). Cohen’s *d* values were also calculated to evaluate the effect sizes for the difference between variables, which was considered small when *d* was between 0.2 and 0.3, medium when *d* was around 0.5, and large when *d* > 0.8 (Cohen, 2013). Data were presented as means (standard deviations, SDs), and the level of *p* < 0.05 was considered statistically significant.

## Results

### Diet Compliance, Dietary Compositions, Daily Physical Activities

Urine ketone was introduced as an indicator for diet compliance. During the ND period, urinary ketosis was only detected on 0.2 ± 0.8% of the days, whereas during the KD intervention, urinary ketosis was detected on 97.7 ± 3.9% of the days, suggesting that the subjects had good compliance with the KD. It should be noted that the days’ (%) urinary ketones during KD were calculated after excluding the data of the three initial transition days.

**FIGURE 2:**
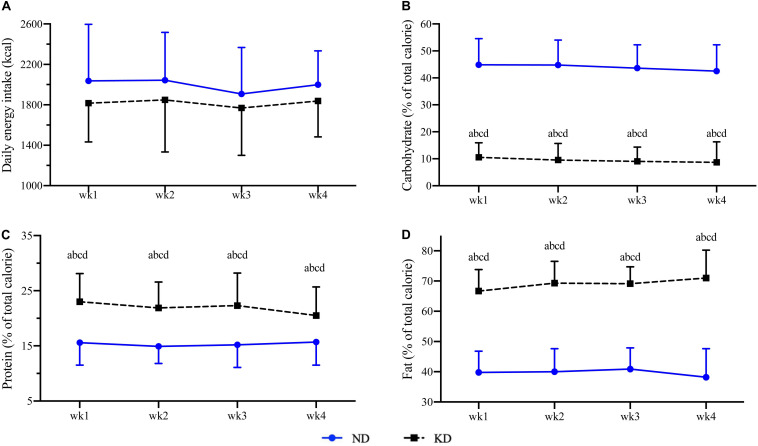
Daily energy intake **(A)**, proportions of carbohydrate **(B)**, protein **(C)**, and fat **(D)** intakes during normal diet (ND) and ketogenic diet (KD). Compared to week 1 of ND at **a** *p* < 0.01; compared to week 2 of ND at **b** *p* < 0.01; compared to week 3 of ND at **c** *p* < 0.01; compared to week 4 of ND at **d** *p* < 0.01.

The mean daily energy intake during the ND period was 1967 ± 362 kcal, of which carbohydrates, proteins, and fats accounted for 44.0 ± 7.6% (217.2 ± 53.3 g), 15.4 ± 3.3% (75.2 ± 20.8 g), 39.6 ± 5.8% (86.3 ± 19.1 g) of daily energy intake (Figure 2). During the KD intervention, the average daily energy intake and the proportions of energy intake derived from carbohydrates, proteins, and fats were 1817 ± 285 kcal, 9.2 ± 4.8% (40.7 ± 21.5 g), 21.9 ± 3.4% (95.4 ± 21.1 g), and 69.0 ± 5.4% (136.4 ± 25.7 g), respectively (Figure 2). No changes in daily energy intake were observed in any of the weeks during ND and KD (*p* > 0.05), whereas the macronutrient compositions were significantly changed during the KD intervention when compared to the ND period with higher proportions of protein (*p* < 0.01) and fat (*p* < 0.01) intake and a lower proportion of carbohydrate intake (*p* < 0.01) during KD (data are presented in Supplementary Table S1).

Daily physical activities were between 7898 and 8954 steps during the ND period and between 7463 and 8346 steps during the KD intervention. There was no statistical difference on daily physical activities among the eight time points measured throughout the study period (data are presented in Supplementary Table S2).

### Changes in Anthropometric Parameters, CRF, and Blood Profiles

After KD intervention, the subjects lost 2.9 ± 2.1 kg of body weight (*p* < 0.01, η^2^ = 0.686) and reduced BMI by 1.1 ± 0.7 kg⋅m^–2^ (*p* < 0.01, η^2^ = 0.702), which remained unchanged during the ND period (Tables 1, 2). The KD intervention also significantly reduced the subjects’ WC (−4.0 ± 3.2 cm, *p* < 0.01, η^2^ = 0.566), HC (−2.5 ± 2.3 cm, *p* < 0.01, η^2^ = 0.554), WHR (−0.02 ± 0.03 cm, *p* < 0.05, η^2^ = 0.218), and percentage of body fat (BF%, −2.0 ± 2.2%, *p* < 0.01, η^2^ = 0.707). No differences in CRF level were found between ND and KD at pre- and post-measurements (*p* > 0.05). Circulating leptin level was significantly decreased in response to KD (*p* < 0.05, η^2^ = 0.370), and the concentrations of ghrelin, TNF-α, and MCP-1 were unchanged after 4 weeks of KD intervention (Table 1). In addition, we found significant correlations between fasting leptin level and body composition variables before ND (*r* = 0.545–0.796, *p* < 0.05), before KD (*r* = 0.510–0.715, *p* < 0.05), and after KD (*r* = 0.480–0.674, *p* < 0.05, Table 3). But, when using the difference values measured before and after the KD intervention, there was no association between KD-induced changes in leptin and body composition (*p* > 0.05). No correlation was found between fasting ghrelin level and body composition variables measured at any time points (*p* > 0.05, Table 3).

**TABLE 1:** Main outcome variables before and after ND and KD.

	**Pre_ND**	**Post_ND (Pre_KD)**	**Post_KD**	**Within-subjects effects**		
				***F***	***p***	**η^2^**
Age (year)	21.0 (3.7)					
Height (cm)	162.1 (5.2)					
Weight (kg)	65.5 (7.7)	65.1 (8.1)	62.1(7.1)^∧∧^**	41.596	0.000	0.686
BMI (kg⋅m^–2^)	24.9 (2.7)	24.8 (2.8)	23.6(2.6)^∧∧^**	44.688	0.000	0.702
WC (cm)	76.9 (7.7)	77.2 (7.7)	73.6(6.0)^∧∧^**	24.763	0.000	0.566
HC (cm)	100.1 (5.0)	100.0 (4.7)	97.4(4.3)^∧∧^**	23.560	0.000	0.554
WHR	0.77 (0.05)	0.77 (0.05)	0.75(0.04)^∧^*	5.298	0.009	0.218
BF%	35.2 (3.8)	34.7 (3.9)	32.2(4.6)^∧∧^*	12.075	0.002	0.707
V̇O_2__peak_ (ml⋅min^–1^)	1.67 (0.24)	1.63 (0.22)	1.57 (0.22)	2.177	0.127	0.103
V̇O_2__peak_ (ml⋅min^–1^⋅kg^–1^)	25.6 (3.9)	25.4 (4.8)	24.5 (3.1)	1.402	0.258	0.069
Leptin (ng⋅ml^–1^)	14.5 (10.1)	11.9 (6.8)	6.9(6.2)^∧∧^**	11.168	0.000	0.370
Ghrelin (pg⋅ml^–1^)	882.0 (703.9)	856.8 (602.7)	785.7 (647.4)	0.535	0.590	0.029
TNF-α (pg⋅ml^–1^)	5.4 (2.0)	5.0 (2.1)	4.6 (1.5)	1.756	0.186	0.085
MCP-1 (pg⋅ml^–1^)	137.9 (44.8)	131.3 (41.8)	122.9 (20.0)	0.919	0.407	0.046

Outcome variables are presented as mean (standard deviation). ND, normal diet; KD, ketogenic diet; BMI, body mass index; WC, waist circumference; HC, hip circumference; WHR, waist-to-hip ratio; BF%, percentage of body fat; V̇O_2__peak_, peak oxygen uptake; TNF-α, tumor necrosis factor-alpha; MCP-1, monocyte chemoattractant protein-1. Comparison to Pre_ND at ^*p* < 0.05, ^^*p* < 0.01, comparison to Post_ND at *p < 0.05, **p < 0.01.

**TABLE 2:** Changes in outcome variables after ND and KD.

	**ND**	**KD**	**ES *(d)***
	**Post_ND – Pre_ND**	**Post_KD – Pre_KD**	
ΔWeight (kg)	−0.4(1.5)	−2.9(2.1)**	1.42
ΔBMI (kg⋅m^–2^)	−0.2(0.6)	−1.1(0.7)**	1.44
ΔWC (cm)	0.7 (1.5)	−4.0(3.2)**	1.91
ΔHC (cm)	−0.1(1.6)	−2.5(2.3)**	1.23
ΔWHR	0.01 (0.02)	−0.02(0.03)**	1.22
ΔBF%	−0.6(1.8)	−2.0(2.2)	0.68
ΔV̇O_2__peak_ (ml⋅min^–1^)	0.0 (0.2)	−0.1(0.2)	0.06
ΔV̇O_2__peak_ (ml⋅min^–1^⋅kg^–1^)	−0.3(3.8)	−0.9(3.3)	0.17
ΔV̇O_2__peak_%	−0.8(14.2)	−1.9(11.5)	0.09
ΔLeptin (ng⋅ml^–1^)	−2.7(7.1)	−5.0(6.0)	0.36
ΔGhrelin (pg⋅ml^–1^)	−25.1(413.3)	−71.1(405.0)	0.11
ΔTNF-α (pg⋅ml^–1^)	−0.4(2.0)	−0.4(1.9)	0.02
ΔMCP-1 (pg⋅ml^–1^)	−6.5(62.1)	−8.4(38.8)	0.04

Outcome variables are presented as mean (standard deviation). ND, normal diet; KD, ketogenic diet; BMI, body mass index; WC, waist circumference; HC, hip circumference; WHR, waist-to-hip ratio; BF%, percentage of body fat; V̇O_2__*peak*_, peak oxygen uptake; TNF-α, tumor necrosis factor-alpha; MCP-1, monocyte chemoattractant protein-1; Delta (Δ), change from pre- to post-intervention. Δ ND vs. Δ KD at **p < 0.01, Cohen’s *d* value for effect size (ES) when compared to Δ ND.

**TABLE 3:** The correlation coefficients between the hormones and body composition.

		**Weight**	**BMI**	**WC**	**HC**	**WHR**	**BF%**
Pre_ND	Leptin	0.615**	0.796**	0.545*	0.668**	0.296	0.664**
	Ghrelin	0.176	0.183	0.137	0.271	–0.001	0.041
Pre_KD	Leptin	0.568**	0.715**	0.587**	0.510*	0.516*	0.531*
	Ghrelin	–0.163	–0.165	–0.176	–0.015	–0.294	–0.409
Post_KD	Leptin	0.523*	0.674**	0.433	0.480*	0.213	0.230
	Ghrelin	–0.189	–0.005	–0.143	–0.095	–0.131	–0.164
Delta_KD	Δ Leptin	0.412	0.439	0.373	0.067	0.344	0.217
	Δ Ghrelin	–0.066	–0.140	–0.020	0.401	–0.271	–0.193

ND, normal diet; KD, ketogenic diet; BMI, body mass index; WC, waist circumference; HC, hip circumference; WHR, waist-to-hip ratio; BF%, percentage of body fat; Delta (Δ), change from pre- to post-intervention of KD. *p < 0.05, **p < 0.01.

### Adverse Events

During the ND period, there was no feedback on any adverse event, but during the KD period, we received 10 complaints about adverse events from seven subjects; these complaints included fatigue (five complaints), constipation (three complaints), reduced appetite (one complaint), and diarrhea (one complaint).

## Discussion

Consistent with findings in Western countries, the present study shows that the 4-week non-calorie-restricted KD dietary approach was also effective in losing weight and reducing abdominal adiposity in Chinese overweight/obese females without impairing their CRF level. In addition, the short-term KD intervention reduced serum leptin concentration, but left unaffected the inflammation biomarkers of TNF-α and MCP-1.

The overweight/obese females lost an average of 2.9 kg of total body mass after the 4-week KD intervention, corresponding to 1.1 unit of BMI. Also, without calorie restriction, the decrement of body mass was similar to some previous studies that reported ∼2.5 kg weight losses in Western adults after 3–6 weeks of KD administration (Sharman et al., 2002; Volek et al., 2002, 2004b; Urbain et al., 2017; Oneal et al., 2019). In contrast, several other KD studies with restricted calorie intake showed greater weight losses (5.0–8.0 kg) in overweight or obese Chinese adults in response to 8 (Gu et al., 2013) and 12 weeks (Liu et al., 2013; Sun et al., 2019) of KD intervention. Given that no previous studies have compared the weight loss effects of energy-restricted KD to non-energy-restricted KD in the same population, it is difficult to know whether calorie restriction has additional benefits in promoting weight loss. Nonetheless, without changing subjects’ habitual calorie intake, the weight reduction after the non-energy-restricted KD intervention in the present study should be mainly ascribed to the changes in macronutrient composition. More than weight loss, we found significant reductions in WC (−4.0 cm, *d* = 191), HC (−2.5 cm, *d* = 1.23), and WHR (−0.02, *d* = 1.22) in the overweight/obese females. This finding supported previous studies that reported that 8 weeks of KD intervention significantly reduced subcutaneous and visceral fat mass in obese but healthy adults (Gu et al., 2013; Valenzano et al., 2019). WC has been recognized as a surrogate indicator of visceral adiposity and is closely associated with cardiometabolic risk (Nazare et al., 2015); thus, marked reductions in WC and WHR may have clinical significance in reducing cardiac and metabolic risks (Neeland et al., 2013; Nazare et al., 2015). Consistently, BF% was decreased by 2.0% as measured using skinfold thickness. These findings illustrate that the KD intervention was not only effective in reducing overall body weight and body fat mass, but it was also beneficial for abdominal fat loss, which is more closely related to cardiometabolic risks.

Higher CRF level is proven to be associated with lower rates of cardiovascular disease events and total mortality (Kodama et al., 2009; Lee et al., 2011; Almallah et al., 2018) regardless of BMI improvement (Lee et al., 2011). However, several studies have reported that KD impaired the CRF level (Okeeffe et al., 1989; Urbain et al., 2017) because the minimal carbohydrate supply during KD intertvention could alter energy metabolism, resulting in reduced muscle glycogen stores and glycolytic-enzyme activities (Chang et al., 2017) as well as central fatigue (Fiorenzo et al., 2020). These metabolic changes limited the energy availability during exercise and, subsequently, impaired the maximal aerobic capacity. Contrary to these studies but supported by others (Brinkworth et al., 2009b; Klement et al., 2013), the CRF level was not changed by the 4-week KD intervention in the present study. These findings suggest that the short-term KD intervention seemed unlikely to affect CRF level, which can be adopted by the overweight/obese Chinese females as a weight-loss diet regime.

An important hypothesis for the mechanism by which KD causes body fat loss could be related to the satiety-increasing effect of higher dietary protein (Weigle et al., 2005; Johnstone et al., 2008). And several studies illustrate that such an effect may be regulated through appetite-mediating hormones, such as leptin and ghrelin (Weigle et al., 2005; Sumithran et al., 2013). Leptin is a hormone mainly synthesized by adipose cells and involved in the regulation of energy balance and fat storage through suppressing hunger (Ahima and Flier, 2000). Previous studies have shown that serum leptin level was declined in parallel with reduced appetite (Weigle et al., 2005; Johnstone et al., 2008; Sumithran et al., 2013) as well as KD-induced reduction in adipose tissue (Boden et al., 2005; Weigle et al., 2005; Sumithran et al., 2013). Although the leptin level was also decreased after KD administration, the KD-induced changes in body composition were not correlated with the alterations in leptin level in the present study, indicating that changes in leptin have less to do with the regulation of body composition during KD. Thus, the decline in the leptin level is more likely a result of the reduced adipose tissue, and there may be other mechanisms rather than leptin pathways that modulate the reductions of body mass and fat mass during KD, for example, pathways relating to fat mobilization. In contrast, the orexigenic hormone, ghrelin, which stimulates appetite, increases food intake and promotes fat storage, was shown to be increased after KD intervention (Boden et al., 2005; Weigle et al., 2005; Sumithran et al., 2013). However, we found a slight but non-significant reduction in ghrelin concentration after KD intervention. Moreover, the daily energy intake was unchanged over the KD period as compared to ND, and this suggests that the overweight/obese females had an unchanged appetite during KD. Therefore, suppressing appetite and spontaneous calorie intake seems to be unable to explain the KD-induced weight reduction and fat mass loss in this study. Other mechanisms, such as alterations in metabolic fuel and insulin level, may also have played important roles in body composition regulation. Under the KD condition in which the carbohydrate intake is drastically reduced, the insufficient metabolic fuel for glucose oxidation forces the body to look for alternative energy sources. Thus, the reliance on metabolic pathways of fat oxidation and gluconeogenesis is increased under KD (Paoli et al., 2013; Ludwig and Friedman, 2014). The main substitute energy source is derived from increased fatty acid oxidation (Paoli et al., 2013). The increased fat oxidation not only promotes the depletion of fat storage in tissues, but also leads to an overproduction of ketone bodies, which can inhibit appetite (Sumithran et al., 2013). Another alternative source of energy is gluconeogenesis from proteins, which is considered an energy-demanding metabolic process that can “waste” an additional 400–600 kcal of calories per day (Fine and Feinman, 2004). Simultaneously, the demand of insulin in assisting glucose uptake is reduced. As a result, fasting insulin level was consistently found to be decreased after KD (Sharman et al., 2002, 2004; Johnstone et al., 2008; Gu et al., 2013; Liu et al., 2013), which further accelerated lipolysis and oxidation of stored and ingested fat (Zammit, 2006). In addition, isocaloric exchange of dietary carbohydrate for fat was found to have decreased respiratory quotient and increase the 24-h energy expenditure in overweight or obese men (Hall et al., 2016). These potential mechanisms may be responsible for the observed losses of body mass and abdominal fat mass in the present study.

Many studies have shown that KD approaches had an anti-inflammatory effect by reducing inflammatory markers, including C-reactive protein, TNF-α, MCP-1, interleukin-6 (IL-6), and IL-8 (Sharman and Volek, 2004; Dansinger et al., 2005; Forsythe et al., 2008; Ratliff et al., 2008), which are generally higher in obesity. However, a 4-week KD intervention did not change proinflammatory cytokines (i.e., C-reactive protein, TNF-α, IL-6) in normal-weight women was also reported (Volek et al., 2003). Consistent with this study, the present study failed to find any influence of KD on the inflammation biomarkers of TNF-α and MCP-1.

By assessing V̇O_2__*peak*_ at pre- and post-measurements, the current study eliminated the concern that KD approaches may impair CRF. Another strength of the present study is that, in addition to the total body mass and overall fat mass, we also assessed changes in abdominal and visceral adiposity in response to KD using surrogate indicators, WC and WHR. Nonetheless, it would be better to assess visceral adiposity accurately using more sophisticated techniques, such as magnetic resonance imaging or dual-energy X-ray absorptiometry. Given that the subjects had difficulties with taking invasive blood ketone tests, we used urinary ketone tests to qualitatively determine whether they were in nutritional ketosis, and this may not be accurate enough. In addition, the intervention duration was relatively short in our study, which limited the interpretation of the long-term weight-loss effects of KD as well as weight maintenance. Finally, this study did not measure the subject’s subjective appetite and hormones relating to fat mobilization, making it hard to interpret possible mechanisms. In this regard, future studies would be benefit from evaluating the long-term weight-loss effects of KD and the underlying mechanisms in different populations.

## Conclusion

Taken together, the 4-week KD intervention led to marked reductions in body mass as well as total and abdominal fat mass without any adverse effect on CRF, suggesting that the KD dietary approaches could also be effective and feasible for the large overweight/obese population in China. Circulating leptin concentration was reduced, but the ghrelin level and energy intake was unchanged in KD. The findings of this study do not seem to support the idea that the weight-loss effect of KD is due to reduced appetite. Further study is required to determine whether the weight-loss effect of KD is mediated through appetite.

## Data Availability Statement

The raw data supporting the conclusions of this article will be made available by the authors, without undue reservation.

## Ethics Statement

The studies involving human participants were reviewed and approved by the Panel on Social Science & Humanities Research Ethics of University of Macau (RC Ref. no. MYRG2017-00199-FED). The patients/participants provided their written informed consent to participate in this study.

## Author Contributions

SS, ZK, and JN: research design. ZK: funding acquisition. SS and ZK: data collection. SS, QS, and HZ: data analysis and interpretation. SS, ZK, and JN: manuscript drafting. SS, ZK, QS, HZ, TT, and JN: manuscript revision. All authors read and approved the final version of the manuscript.

## Conflict of Interest

The authors declare that the research was conducted in the absence of any commercial or financial relationships that could be construed as a potential conflict of interest.
