# Supplementary material for: Short-Term Ketogenic Diet Improves Abdominal Obesity in Overweight/Obese Chinese Young Females
Source: Front Physiol. 2020 Jul 28;11:856. doi: 10.3389/fphys.2020.00856 (PMC7399204; doi:10.3389/fphys.2020.00856)
Supplement: Supplementary file 1 [file Data_Sheet_1.PDF]

Table S1. Energy intake and macronutrient compositions during ND and KD

	Week 1	Week 2	Week 3	Week 4
Energy intake (kcal)				
ND	2037 (559)	2043 (474)	1907 (460)	1999 (335)
KD	1816 (383)	1848 (514)	1768 (467)	1838 (355)
Carbohydrate (% of energy intake)				
ND	44.9 (9.7)	44.8 (9.2)	43.6 (8.7)	42.5 (9.8)
KD	10.5 (5.4) ^{abcd}	9.5 (6.2) ^{abcd}	9.0 (5.3) ^{abcd}	8.7 (7.6) ^{abcd}
Fat (% of energy intake)				
ND	39.8 (7.0)	40.0 (7.6)	40.9 (7.0)	38.2 (9.4)
KD	66.7 (7.1) ^{abcd}	69.3 (7.2) ^{abcd}	69.1 (5.6) ^{abcd}	71.0 (9.2) ^{abcd}
Protein (% of energy intake)				
ND	15.6 (4.1)	14.9 (3.1)	15.2 (4.1)	15.7 (4.2)
KD	23.0 (5.1) ^{abcd}	21.9 (4.7) ^{abcd}	22.3 (5.9) ^{abcd}	20.5 (5.2) ^{abcd}
Carbohydrate (in g)				
ND	229.8 (80.5)	229.4 (70.4)	210.1 (74.9)	210.4 (52.4)
KD	48.2 (27.6) ^{abcd}	38.2 (23.7) ^{abcd}	40.1 (26.9) ^{abcd}	39.5 (35.1) ^{abcd}
Fat (in g)				
ND	90.2 (28.9)	90.2 (24.2)	85.1 (18.4)	85.5 (27.2)
KD	133.9 (34.9) ^{aBcd}	127.8 (44.8) ^{aBcd}	137.0 (43.2) ^{abcd}	145.6 (38.9) ^{abcd}
Protein (in g)				
ND	77.7 (21.6)	76.5 (24.1)	73.1 (27.0)	77.9 (21.6)
KD	103.3 (27.3) ^{ABcd}	89.2 (33.4)	95.2 (27.1)	93.1 (27.7)

Outcome variables are presented as mean (standard deviation). ND: normal diet, KD: ketogenic diet. Compared to week 1 of ND at ^{A} $p < 0.05$, ^{a} $p < 0.01$; compared to week 2 of ND at ^{B} $p < 0.05$, ^{b} $p < 0.01$; compared to week 3 of ND at ^{c} $p < 0.01$; compared to week 4 of ND at ^{d} $p < 0.01$.
