# Supplementary material for: Short-Term Ketogenic Diet Improves Abdominal Obesity in Overweight/Obese Chinese Young Females
Source: Front Physiol. 2020 Jul 28;11:856. doi: 10.3389/fphys.2020.00856 (PMC7399204; doi:10.3389/fphys.2020.00856)
Supplement: Supplementary file 2 [file Data_Sheet_2.PDF]

Table S2. Daily physical activities during ND and KD

Steps	Week 1	Week 2	Week 3	Week 4
ND	8193 (2844)	8138 (2821)	8954 (1620)	7898 (2295)
KD	7463 (2236)	8246 (2775)	8346 (2906)	8046 (2028)

Outcome variables are presented as mean (standard deviation). ND: normal diet, KD: ketogenic diet.
